# Memories of a Grandparent’s Death: Preparation for Future Losses

**DOI:** 10.1093/geroni/igaa057.2050

**Published:** 2020-12-16

**Authors:** Cory Bolkan, Raven Weaver

**Affiliations:** 1 Washington State University, Vancouver, Washington, United States; 2 Washington State University, Pullman, Washington, United States

## Abstract

Experiences of death in early life may result in identity-defining memories that last a lifetime. Autobiographical memories serve psychosocial functions, acting as guides for future behavior. Understanding early death experiences may thus inform lifelong personal views about death, dying, and bereavement. We queried 50 adults (ages 19 – 67 years) using a structured set of questions to recall and write about their earliest and most significant losses. The narratives were qualitatively analyzed using the constant comparative method associated with grounded theory. Results indicated a grandparent’s death was the most frequently reported significant loss, reflecting the value of intergenerational relationships and the long-lasting impact of grandparent death. Themes also emerged concerning participants’ reports of the benefits of actively remembering and reflecting on loss, as well as learning from others’ losses, which further deepened their views of death. These findings highlight how early memories of death, including one's grandparents, can have lifelong impact.

